# Unveiling the Mammalian Diversity and Conservation Significance of Jianfengling Region: A Camera-Trapping Survey of Mammals in Hainan Tropical Rainforest National Park

**DOI:** 10.3390/ani16050721

**Published:** 2026-02-25

**Authors:** Wenbo Yan, Xiangxiang Lu, Zhigao Zeng, Shaoliang Xue, Qi Wang, Shiqin Mo, Chunshen Liang

**Affiliations:** 1 School of Biological Science and Engineering, Shaanxi University of Technology, Hanzhong 723001, China; luxx2906441543@163.com (X.L.); wangqis6@163.com (Q.W.); 2 Hainan Institute of National Park, Haikou 571100, China; 3 State Key Laboratory of Animal Biodiversity Conservation and Integrated Pest Management, Institute of Zoology, Chinese Academy of Sciences, Beijing 100101, China; 4 Jianfengling Branch of the Management Office of Hainan Tropical Rainforest National Park, Ledong 572542, China; xues1415294984@163.com (S.X.); mos1113424741@163.com (S.M.); l13876722005@126.com (C.L.)

**Keywords:** camera trap, Endangered species, mammalian diversity, occupancy model, Hainan Island

## Abstract

The Jianfengling region on southwestern Hainan Island is a sanctuary for a diverse range of wild animals. However, the exact extent of mammal species diversity and conservation status in Jianfengling remains largely unknown. Using camera-trapping data spanning from October 2020 to November 2021, this study focused on the diversity and distribution status of mammal species in Jianfengling, Hainan Tropical Rainforest National Park, China. The survey, with 41,571 camera days and 8091 independent detections, revealed 15 mammalian species belonging to 6 orders and 10 families. Among these detected species, one was categorized as Critically Endangered, and one as Near Threatened on the IUCN Red List. According to the Red List of China’s Vertebrates, one was categorized as Critically Endangered, one as Endangered, three as Vulnerable, and three as Near Threatened. Additionally, one was designated China’s national first-class key protected wildlife, and five were designated China’s national second-class key protected wildlife. Notably, populations of the Chinese pangolin (*Manis pentadactyla*) were confirmed to persist in the wild of Jianfengling. Occupancy modeling results showed that the occupancy of Rhesus monkey (*Macaca mulatta*), Hainan muntjac (*Muntiacus nigripes*), common palm civet (*Paradoxurus hermaphroditus*), and Mainland leopard cat (*Prionailurus bengalensis*) increased with increasing elevation. Vegetation type would only affect the occupancy of the Rhesus monkey. Normalized Difference Vegetation Index and anthropogenic activities had no obvious effect on the occupancy of mammals. In terms of the relative abundance index (RAI) of mammals captured by camera traps, the most prevalent species identified was the Asiatic brush-tailed porcupine (*Atherurus macrourus*), followed by the wild boar (*Sus scrofa*), Pallas’s squirrel (*Callosciurus erythraeus*), and Hainan muntjac. The monitoring also captured a significant number of images of domestic dogs and humans. These findings establish a baseline for species richness and occupancy of mammals in a typical island tropical rainforest in Hainan Tropical Rainforest National Park. We hope that our findings could be used to assess the effectiveness of conservation monitoring programs in the coming years, as species richness surveys provide an important baseline for conservation planning.

## Simple Summary

Based on a camera-trapping survey (41,571 camera days) in Jianfengling, Hainan Tropical Rainforest National Park (2020–2021), 15 wild mammal species from 6 orders and 10 families were recorded. The persistence of the Critically Endangered Chinese pangolin (*Manis pentadactyla*) was confirmed. Occupancy modeling indicated that species like the Rhesus monkey (*Macaca mulatta*) and Hainan muntjac (*Muntiacus nigripes*) showed higher occurrence at higher elevations. The Asiatic brush-tailed porcupine (*Atherurus macrourus*) had the highest relative abundance. This study establishes a crucial biodiversity baseline for conservation monitoring in this island tropical rainforest.

## 1. Introduction

As biodiversity hotspots, tropical forests play a pivotal role in global species conservation by harboring an estimated 90% of the world’s species, covering just 6% of the Earth’s land surface [1,2,3]. The historical coverage of tropical rainforests was greater than 90% on Hainan Island; this coverage decreased to 25.5% in 1956 [4]. Owing to urbanization, logging activity, and the rapid development of tropical agriculture, tropical rainforests have experienced rapid loss and degradation, with the area reaching only 4% of Hainan Island by the end of the 20th century [4]. In recent years, increasing anthropogenic disturbances, including tropical crop implantation and ecotourism development, have exacerbated habitat loss and fragmentation in local wildlife, and even poaching activities have threatened the survival of certain species [5,6].

Mammals are a crucial component of forest ecosystems and play a significant role in ecosystem services and functions such as nutrient cycling, plant growth, and seed dispersal owing to their life cycles and reproductive strategies [7,8,9,10]. However, they are also among the most threatened animal groups, facing issues such as habitat loss, overexploitation, climate change, and invasive species [11].

Owing to their dense vegetation and complex community structures, tropical rainforests provide suitable microhabitats for various mammals, but their visibility in tropical rainforests is limited [12]. Many mammal populations have low densities, secretive behaviors, and nocturnal activity patterns in tropical rainforests [13]. Therefore, one of the primary challenges in researching and protecting tropical rainforest mammals lies in the considerable difficulty associated with detecting and monitoring them. Camera-trapping technology, a method that offers advantages such as long-term, continuous, covert, and minimally disruptive monitoring, is an economical, efficient, and easy-to-use “noninvasive” tool suitable for studying forest wildlife mammals that are rare, secretive, and nocturnal [14,15,16]. This technology aids in the study of mammal diversity, distribution, and activity patterns [17,18,19,20,21].

Located southwest of Hainan Island, Jianfengling stands as one of the best-preserved tropical forest areas on the island, with rich biodiversity (having 6883 species) [22]. To protect the tropical rainforest ecosystem and its biodiversity in Jianfengling, China established the Hainan Jianfengling Nature Reserve in 1956, and in 2020, the Jianfengling Branch of Hainan Tropical Rainforest National Park was formally inaugurated. Historical records indicate that Jianfengling is home to 68 species of mammals, including the clouded leopard (*Neofelis nebulosa*), the Hainan gibbon (*Nomascus hainanus*), Eld’s deer (*Rucervus eldii*), Asian black bears (*Ursus thibetanus*), and the Chinese pangolin (*Manis pentadactyla*) [23]. However, Hainan gibbon and Eld’s deer vanished from Jianfengling after the 1980s [24,25], whereas clouded leopards and Asian black bears experienced island-wide extirpation after the 1990s [5,6,26]. Apex predator functional extinction could result in Empty Forest Syndrome with structural disequilibrium extant fauna community [27]. However, the exact extent of mammal species diversity and conservation status in Jianfengling remains largely unknown due to the lack of baseline data necessary for systematic monitoring, surveys, and management. There is no systematic research or reliable estimates of their density and abundance. Therefore, identifying mammal species in Jianfengling and implementing protective measures are essential for preserving the ecological integrity and cultural heritage of the region.

In this study, we concentrated on researching the diversity and distribution of mammal species in Jianfengling, Hainan Tropical Rainforest National Park, China. We aimed to address the following two scientific questions.

1. What constitutes the present assemblage and conservation standing of the mammalian community within the Jianfengling region? In particular, do populations of critically rare and Endangered species, such as the Chinese pangolin, continue to endure?
2. In light of the functional extinction of apex predators, which environmental determinants (for instance, elevation, anthropogenic disturbance, and vegetation type) govern the spatial distribution and occupancy of the residual mammalian species?

## 2. Materials and Methods

### 2.1. Study Area

**Figure 1:**
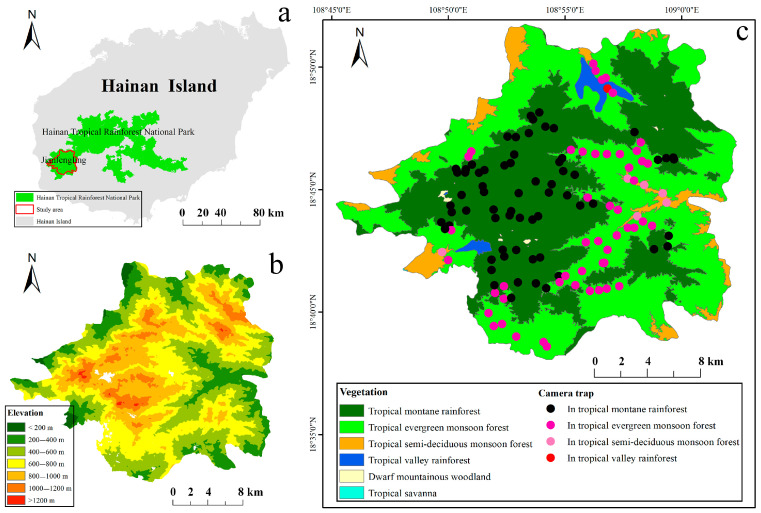
(**a**) Study area and Hainan Tropical Rainforest National Park, China. (**b**) Digital elevation model (DEM) derived from 1:1 million data provided by the National Catalog Service for Geographic Information (https://www.webmap.cn/main.do?method=index, accessed on 10 October 2024), and (**c**) vegetation type derived from 1:100 thousand land use type data provided by the Jianfengling Branch Bureau of Hainan Tropical Rainforest National Park Management Office. The inset dots represent the locations of the camera traps, and different colors represent camera traps located in different vegetation types.

Jianfengling, located southwest of Hainan Island, lies within the Jianfengling Branch of Hainan Tropical Rainforest National Park (108°45′–109°03′ E, 18°38′–18°52′ N; Figure 1a). This territory is predominantly populated by the Li ethnic group (69.6% of the total population). It covers an area of 678 km^2^, with an elevation range of 112–1412 m above mean sea level (Figure 1b). It has a tropical monsoon climate and distinct dry and rainy seasons. The annual average rainfall is 2265.8 mm, with the annual precipitation concentrated between May and October [22]. The annual average temperature is 24.5 °C, with rainfall and heat occurring during the same period [22]. Its unique ecological environment supports a variety of vegetation types, mainly comprising tropical montane rainforest, tropical evergreen monsoon forest, tropical valley rainforest, and tropical semi-deciduous monsoon forest, accounting for 45.96%, 46.53%, 1.58% and 5.77% of the total area, respectively.

### 2.2. Camera Trapping

Camera-trapping surveys were conducted from October 2020 to November 2021 to monitor mammals in Jianfengling via 123 camera-trap sites. Sites were selected based on the area ratio of vegetation type: sixty-six sites were distributed in tropical montane rainforests, fifty in tropical evergreen monsoon forests, six in tropical semi-deciduous monsoon forests, and one in tropical valley rainforests, respectively (Figure 1c). Sites were at least 500 m apart to ensure independence among detections between sites [28]. The elevation of these sites ranged from 237 to 1098 m above mean sea level. Each site had one passive infrared camera (E3H series, Shenzhen Ereagle Technology Co., Ltd., Shenzhen, China; Ltl 6511 series, Zhuhai Ltl Acorn Electronics Co., Ltd., Zhuhai, China). Each camera was configured to operate continuously for 24 h. Each event was registered by three photographs followed by a 10–20 s video, with a 1 s interval before the next trigger. Owing to the shooting effect being easily affected by factors such as wind and changes in light intensity, the sensitivity of passive infrared detection was registered as moderate. The cameras were mounted 0.5–1.0 m above ground level, based on animal body size and avoiding weed cover. The camera lenses were adjusted to be parallel to the ground, with a broad view to avoid direct sunlight. Camera traps were strategically positioned in areas of presumably high wildlife activity, animal trails, ridge tops, and fallen logs. These cameras were checked for working conditions every 3 months.

### 2.3. Data Analysis

The method of data processing used involved identifying mammalian species in images and videos, analyzing the data to estimate the frequency of animal occurrence, and combining a geographic information system (GIS) to analyze the distribution of these species [29]. The identification of mammalian species in the images and videos followed the morphological characteristics criteria of Smith and Xie [30], Jiang et al. [31], and Wei et al. [32].

The conservation status of mammals was assessed at both the global and national levels according to the threat categories assigned to the IUCN Red List (http://www.iucnredlist.org/, accessed on 10 October 2025) and the Red List of China’s Vertebrates [33]. The survey effort was quantified by the total number of camera-days, where every 24 h of continuous operation of an infrared camera in the field counts as one camera-day. A single capture event was defined as any sequence for a given species taken more than 30 min after the previous sequence of that species at the same location [34,35,36]. Additionally, we tallied the total number of independent capture events for each species and determined the site occupancy (SO) that recorded each species [35,37]. The SO were calculated via the following formulae:

(1) $$SO=\frac{S}{T}\times 100\%$$

where ‘S’ is the number of camera-trap sites that recorded the species; ‘T’ represents the total number of camera-trap sites; the higher SO value suggested the greater distribution.

The relative abundance index (RAI) was also calculated according to the methods suggested by O’Brien et al. [14] as a species quantitative index to assess the abundance of species within the territory. The RAI values were calculated via the following formula:

(2) $$RAI=\frac{Ai}{N}\times 1000$$

where ‘Ai’ represents the number of independent mammal photographs of the i-th species, and ‘N’ is the total number of camera-trap days; the higher RAI value suggested the greater relative abundance.

We tested both environmental and anthropogenic covariates to evaluate their influence on mammal occupancy rate. Elevation was extracted from a digital elevation model of 1:1 million data provided by the National Catalog Service for Geographic Information (https://www.webmap.cn/, accessed on 10 October 2024) and was included because elevation had been known to influence species distribution and occupancy [38]. Vegetation type and Normalized Difference Vegetation Index (NDVI) were used as vegetation covariates on occupancy [39]. Vegetation type was derived from 1:100 thousand landuse type data provided by the Jianfengling Branch Bureau of Hainan Tropical Rainforest National Park Management Office. NDVI was derived from China regional 250 m normalized difference vegetation index data set (2000–2024) (https://data.tpdc.ac.cn/zh-hans/data/10535b0b-8502-4465-bc53-78bcf24387b3, accessed on 10 May 2025). We used the human footprint index (HFI) as the anthropogenic covariate on occupancy [40]. HFI was derived from the 2020 Global Human Footprint Data released by the Wildlife Conservation Society.

Occupancy modeling was constructed using the “unmarked” package (version 1.5.0) in R (version 4.5.0) software [41]. Detection history was established for animals every 10 days [41]. For any images captured during each segment, 1 indicated “detected”, 0 indicated “not detected”, and NA indicated a camera trap malfunction [41]. Due to the limited sample size, the detection probability was held constant. For each species, we tested various models using different combinations of elevation, vegetation type, NDVI, and HFI covariates (Table S1). These models were ranked using Akaike’s Information Criterion (AIC), and the model with the lowest AIC was considered the most parsimonious model [41]. We then used the most parsimonious model to evaluate species habitat preferences. The model results for evaluating the yellow-bellied weasel (*Mustela kathiah*) and black giant squirrel (*Ratufa bicolor*) did not have enough 0/1 detections to run the occupancy analysis.

## 3. Results

### 3.1. Mammalian Diversity in Jianfengling

Among the 123 camera traps, 9 failed to generate usable data due to loss. Over 41,571 camera days, a total of 151,192 photos and videos were captured. There were 8091 independent detections for mammalian species. There were 832 rodent images of unspecified species because of unclear morphological characteristics. There were 105 domestic dog images and 82 human images. In total, 15 species of mammals belonging to 6 orders and 10 families were identified: Rhesus monkey (*Macaca mulatta*), Chinese pangolin, common palm civet (*Paradoxurus hermaphrodites*), Mainland leopard cat (*Prionailurus bengalensis*), yellow-bellied weasel, Small-toothed ferret badger (*Melogale moschata*), Hainan muntjac (*Muntiacus nigripes*), wild boar (*Sus scrofa*), Northern treeshrew (*Tupaia belangeri*), Malayan porcupine (*Hystrix brachyuran*), Asiatic brush-tailed porcupine (*Atherurus macrourus*), black giant squirrel, Pallas’s squirrel (*Callosciurus erythraeus*), red-hipped squirrel (*Dremomys pyrrhomerus*) and swinhoe’s striped squirrel (*Tamiops swinhoei*) (Table 1).

**Figure 2:**
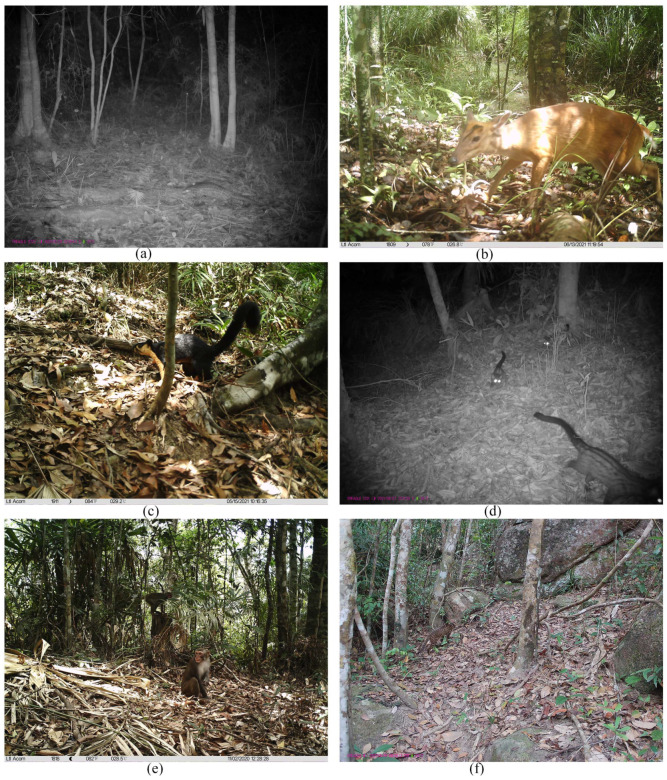
Infrared camera trap images of (**a**) Chinese pangolin, (**b**) Hainan muntjac, (**c**) black giant squirrel, (**d**) common palm civet, (**e**) Rhesus monkey, and (**f**) Mainland leopard cat at the Jianfengling Branch of Hainan Tropical Rainforest National Park, from October 2020 to November 2021.

Among these detected species, one was categorized as Critically Endangered, and one as Near Threatened on the IUCN Red List. According to the Red List of China’s Vertebrates, one was categorized as Critically Endangered, one as Endangered, three as Vulnerable, and three as Near Threatened. Additionally, one was designated China’s national first-class key protected wildlife, and five were designated China’s national second-class key protected wildlife. Notably, we detected four species that are rare in China: Chinese pangolin, common palm civet, Hainan muntjac, and black giant squirrel (Figure 2).

### 3.2. Mammalian Distribution in Jianfengling

**Figure 3:**
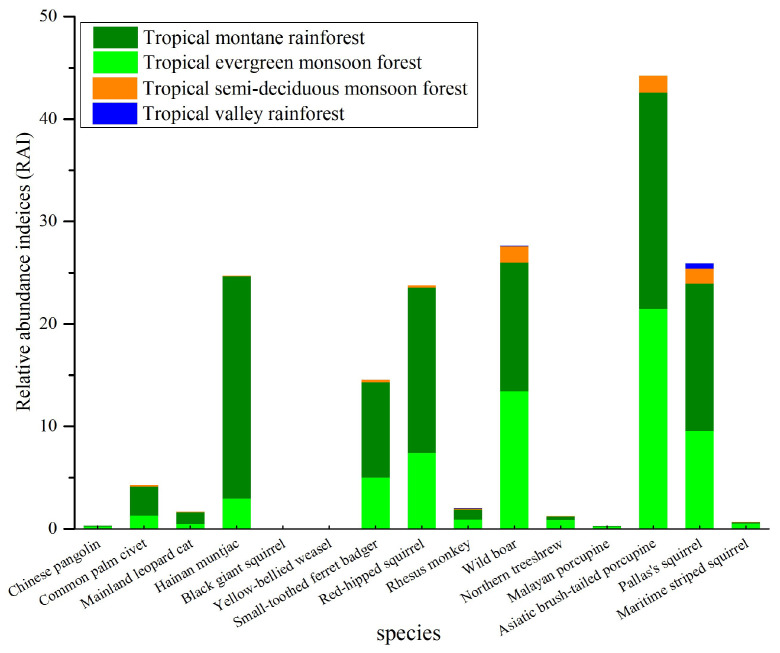
The relative abundance indices (RAI) of species are depicted, with different colors in the columns representing the percentage of traps recording species across various habitat types in the Jianfengling Branch of Hainan Tropical Rainforest National Park, from October 2020 to November 2021.

**Figure 4:**
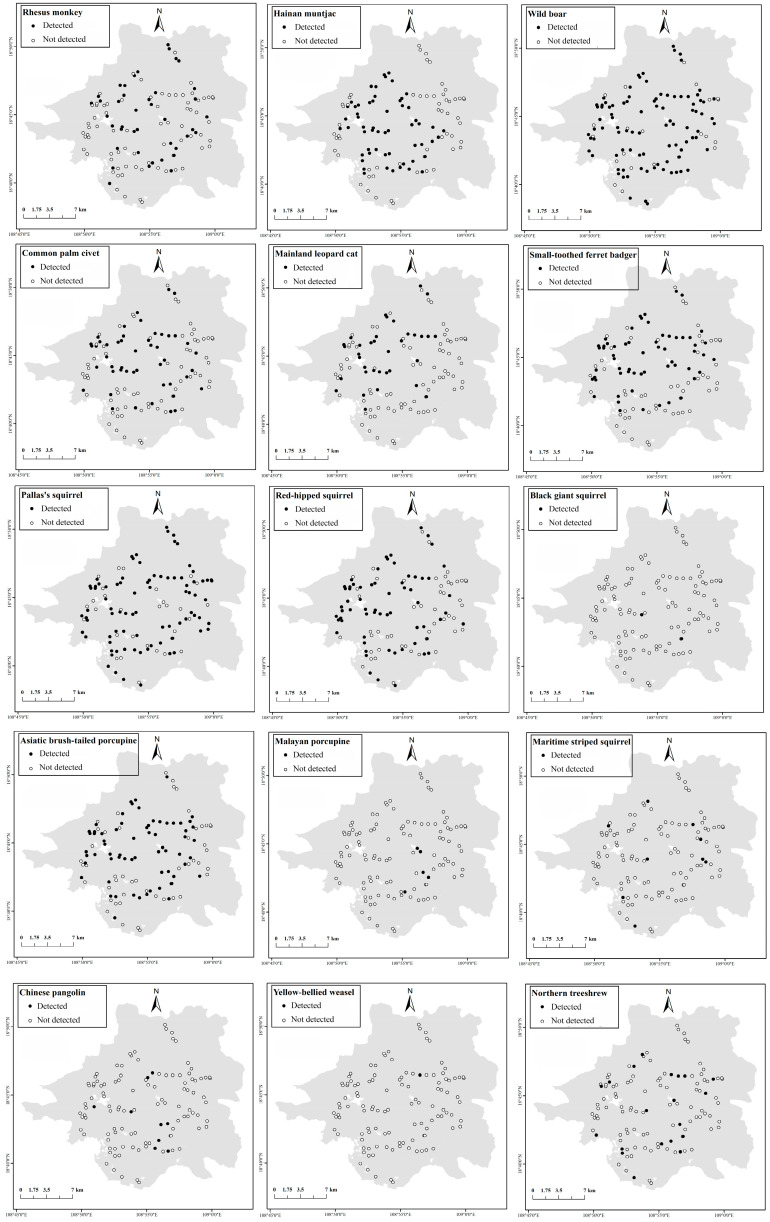
The spatial distribution of different species in the Jianfengling Branch of Hainan Tropical Rainforest National Park, from October 2020 to November 2021.

Rhesus monkey, wild boar, and Pallas’s squirrel were found across all the vegetation types. By contrast, 12 other species only occurred in some specific vegetation (Table 1; Figure 3). There were five species with SO values greater than 50.0%, namely wild boar, Pallas’s squirrel, Asiatic brush-tailed porcupine, red-hipped squirrel, and Small-toothed ferret badger. There were five species with SO values less than 10.0%, namely the Chinese pangolin, black giant squirrel, yellow-bellied weasel, Malayan porcupine, and swinhoe’s striped squirrel (Table 1; Figure 4).

Occupancy modeling results showed that occupancy of Rhesus monkey, Hainan muntjac, common palm civet, and Mainland leopard cat increased with increasing elevation (Table 2). Elevation had no obvious effect on occupancy of Small-toothed ferret badger, Wild boar, Northern treeshrew, Malayan porcupine, Asiatic brush-tailed porcupine, and red-hipped squirrel (Table 2). Vegetation type had an effect on the occupancy of Rhesus monkey, but no effect on the occupancy of other species (Table 2). NDVI and HFI had no obvious effect on occupancy of mammals (Table 2). Chinese pangolin, Pallas’s squirrel, and swinhoe’s striped squirrel could not build occupancy modeling with elevation, vegetation type, NDVI and HFI.

### 3.3. Mammalian Relative Abundance in Jianfengling

The most prevalent species, indicated by the total number of independent capture events and the relative abundance within the entire monitoring area, was the Asiatic brush-tailed porcupine (Figure 3). In terms of the RAI, the second most abundant species was wild boar, with an RAI of 27.62, followed by Pallas’s squirrel, with an RAI of 25.91, and Hainan muntjac, with an RAI of 24.70. Conversely, the species with the lowest relative abundance recorded with no more than fifteen independent capture events were the Chinese pangolin, black giant squirrel, yellow-bellied weasel, and Malayan porcupine, each having an RAI value of less than 0.30 (Figure 3). Additionally, monitoring also captured a significant number of domestic dogs (RAI of 2.53), as well as human disturbances (RAI of 1.97) such as tea picking and medicinal herb collection, even hunting.

## 4. Discussion

This study used camera trapping across Jianfengling to identify 15 mammal species across 6 orders and 10 families within the study area, including several rare and Endangered species, such as Chinese pangolin, common palm civet, Hainan muntjac, and black giant squirrel. Compared with other areas within Hainan Tropical Rainforest National Park [42,43], this finding highlights the diverse mammal community in Jianfengling and demonstrates that Jianfengling holds significant potential for mammal conservation.

Notably, our research confirmed the existence of wild Chinese pangolin populations in Jianfengling. As the principal habitat of the Hainan subspecies of Chinese pangolin [44], the insular population experienced a precipitous decline due to poaching and habitat fragmentation, with sightings of live specimens becoming exceedingly rare by 2008 [45]. While community interviews in 2015 suggested its residual distribution within Hainan Tropical Rainforest National Park [46], tangible field evidence remained conspicuously absent [47]. A pivotal breakthrough occurred from 2015 to 2018, when camera trapping surveys in Jianfengling’s core zones successfully documented pangolin activities [5,6]. Compared to Mo’s results that three years of monitoring only captured two independent photographic records at one site [6], current investigations have revealed significant advancements: the SO of pangolin species in Jianfengling has reached 7.89%, with 12 independent photographic records, including inaugural evidence from peripheral forest zones. This might be attributed to the intensification of conservation measures (ranger patrols and anti-poaching activities) following the formal establishment of the Jianfengling Branch of Hainan Tropical Rainforest National Park in 2020. These findings underscore the crucial conservation value for maintaining genetic diversity, ensuring population stability, and facilitating the ecological restoration of this insular pangolin subspecies.

Phylogenetic analysis supports the recognition of Hainan muntjac as a distinct species within the genus *Muntiacus* [48]. This research sheds light on Hainan muntjac conservation status and habitat requirements. Following Hainan Island’s biogeographic isolation after the late Pleistocene, this distinctive evolutionary crucible fostered exceptional speciation and subspecific differentiation [49]. As a typical island ecosystem, the island not only serves as the type locality for the Hainan subspecies of common palm civet [50], but our current investigation has methodically documented this taxon’s extensive spatial distribution in Jianfengling. Notably, the Hainan subspecies of Rhesus monkey was shaped through prolonged adaptive evolution [51], demonstrating remarkable ecological adaptive capacities across Jianfengling’s elevational spectrum—from valley rainforests to montane forests.

As the biogeographic nucleus of Hainan Island’s tropical forest ecosystems, Jianfengling’s superlative ecological milieu establishes it as a critical sanctuary for numerous rare and Endangered species. Our camera trapping data reveal that this area sustains emblematic examples of flagship species guilds, and six mammal taxa have been systematically documented [52], including the Chinese pangolin, common palm civet, Hainan muntjac, Rhesus monkey, Mainland leopard cat, and black giant squirrel. Comparative analysis against existing biodiversity baseline data from other park sectors demonstrated that Jianfengling’s mammalian community is highly rich, accentuating its exceptional role as an insular ecological refuge sustaining evolutionary distinctiveness [42,43].

Apex predator functional extinction could result in structural disequilibrium of the extant fauna community in Jianfengling. Compared with 20th-century biodiversity baselines, our survey failed to document critical species, including clouded leopards, the Asian black bear, the Hainan gibbon, and Eld’s deer [23]. These absent taxa predominantly occupy high trophic strata (Primates, Carnivora, large Artiodactyla). This ecological void has precipitated marked trophic cascades: extant wild boars and Asiatic brush-tailed porcupines demonstrate extraordinary RAIs of 27.62 and 44.21, respectively, and their demographic explosions are potentially linked to apex predator functional extinction [53]. Comparative analyses have confirmed strong positive correlations between megafaunal population collapse and habitat fragmentation in East Asian tropical forests [54,55,56,57]. The structural disequilibrium within Jianfengling’s extant fauna community epitomizes insular manifestations of Empty Forest Syndrome, revealing cascading consequences of defaunation in relict ecosystems [27].

The HFI had no obvious effect on mammal occupancy in Jianfengling, likely because of a spatial-scale mismatch between the HFI data and our camera-trap survey [40]. Despite this, our research identifies three mechanisms by which anthropogenic disturbance might influence mammalian biodiversity in Jianfengling: poacher incursions (wielding hunting rifles), tourism intensification (peaking at 2500 daily visitors), and community activities (notably free-ranging domestic dogs), collectively manifesting an RAI of 4.50. In particular, 31 camera trap vandalism incidents were recorded during the study period; events of vandalism were more frequent where armed intruders were observed, underscoring the encroachment intensity of illegal wildlife trade networks into protected areas [46]. Historical ethnological research reveals how post-ban cultural (after 1980s) inertia in Li ethnic hunting traditions synergizes with habitat homogenization from ecotourism development, forging complex human–wildlife conflict matrices [4]. This survey did not detect mammals that prefer to inhabit streams and surrounding habitats, such as the oriental small-clawed otter (*Aonyx cinerea*), Javan mongoose (*Herpestes javanicus*), and crab-eating mongoose (*Herpestes urva*). The reason might be that ecotourism along the Tianchi artificial lake and streams has led to a decline in their population, or even their extinction. These patterns are corroborated by monitoring gaps in Mo et al.’s 2019–2021 datasets [5,6].

In Jianfengling, elevation could emerge as a more critical environmental gradient than vegetation type in governing the spatial distribution of various mammalian species. Elevation could mediate species distribution indirectly through its influence on factors such as temperature, humidity, food resource availability, and the intensity of human disturbances [38]. Although the study area comprises four principal vegetation types, they exerted no significant effect on the distribution patterns of the majority of species examined. This implied that the microhabitat variations provided by these vegetation types were insufficient to act as key determinants in restricting their distribution. It is noteworthy that certain rare species could not be integrated into the occupancy model analysis due to insufficient detection frequencies. Thus, the specific influences of elevation and vegetation on these Critically Endangered species await clarification through more prolonged or intensive monitoring initiatives.

## 5. Conclusions

While our study substantially advances the understanding of mammalian diversity and distribution, certain limitations remain. The absence of comparisons with other area data of Hainan Tropical Rainforest National Park restricts insights into how Jianfengling’s mammalian community is highly rich. Furthermore, the camera deployment scheme in the present study might have led to an underestimation of arboreal mammalian diversity and distribution. Despite these limitations, our research establishes a baseline for species richness and occupancy of mammals in a typical island tropical rainforest in Hainan Tropical Rainforest National Park. We hope that our findings can be used to assess the effectiveness of conservation monitoring programs in the coming years, as species richness surveys provide an important baseline for conservation planning. We recommend following the recommendations for mammal conservation in Jianfengling.

1. Based on the low SO values observed for the Chinese pangolin and the black giant squirrel, we recommend establishing strict protection areas within the specific grids where they were detected.
2. In response to the incidents of camera trap vandalism, we recommend enhancing public awareness campaigns and strengthening the intensity and targeting of routine patrols in affected areas.
3. Given the high RAI of wild boars and Asiatic brush-tailed porcupines, combined with the absence of top predators, it is recommended to conduct population dynamics monitoring to assess their potential downward effects on the ecosystem.
4. For future work, we recommend species distribution modeling for the species in this area to better understand their spatial distributions and expand the study coverage to other areas of Hainan Tropical Rainforest National Park.

## Figures and Tables

**Table 1:** Mammalian species were monitored through camera traps at the Jianfengling Branch of Hainan Tropical Rainforest National Park, China, from October 2020 to November 2021. Species status with IUCN and China red list (CR: Critically Endangered; EN: Endangered; VU: Vulnerable; NT: Near Threatened; LC: Least Concern; NE: Not Evaluated), and China national protected category (I: China national first-class key protected wildlife; II: China national second-class key protected wildlife). Information on elevation range observed, vegetation (E: tropical evergreen monsoon forest; M: tropical montane rainforest; S: tropical semi-deciduous monsoon forest; V: tropical valley rainforest). The number of independent capture events, site occupancy (SO), and relative abundance index (RAI).

Species (by Order)	IUCN Red List Status	China’s Vertebrate Red List Status	China’s National Protected Category	Elevation Range Observed (m)	Vegetation Types	Total No. of Independent Capture Events	Site Occupancy (SO)	Relative Abundance Index (RAI)
**Primates**								
Cercopithecidae								
Rhesus monkey *Macaca mulatta*	LC	LC	II	270–1077	E, M, S, V	84	35.96%	2.02
**Pholidota**								
Manidae								
Chinese pangolin *Manis pentadactyla*	CR	CR	I	454–974	E, M	12	7.89%	0.29
**Carnivora**								
Viverridae								
Common palm civet *Paradoxurus hermaphroditus*	LC	EN	II	237–1077	E, M, S	177	42.11%	4.26
Felidae								
Mainland leopard cat *Prionailurus bengalensis*	LC	VU	II	237–1077	E, M	69	30.7%	1.66
Mustelidae								
Yellow-bellied weasel *Mustela kathiah*	LC	NT	/	464	E	1	0.88%	0.02
Small-toothed ferret badger *Melogale moschata*	LC	NT	/	269–1077	E, M, S	604	51.75%	14.53
**Artiodactyla**								
Cervidae								
Hainan muntjac *Muntiacus nigripes*	NE	VU	II	310–1077	E, M, S	1027	50.0%	24.70
Suidae								
Wild boar *Sus scrofa*	LC	LC	/	237–1077	E, M, S, V	1148	82.46%	27.62
**Scandentia**								
Tupaiidae								
Northern treeshrew *Tupaia belangeri*	LC	LC	/	269–956	E, M, S	52	17.54%	1.25
**Rodentia**								
Hystricidae								
Malayan porcupine *Hystrix brachyura*	LC	LC	/	441–683	E, M	11	5.26%	0.26
Asiatic brush-tailed porcupine *Atherurus macrourus*	LC	LC	/	237–1098	E, M, S	1838	57.89%	44.21
Sciuridae								
Black giant squirrel *Ratufa bicolor*	NT	VU	II	564–883	E, M	2	1.75%	0.05
Pallas’s squirrel *Callosciurus erythraeus*	LC	LC	/	237–1077	E, M, S, V	1077	74.56%	25.91
Red-hipped squirrel *Dremomys pyrrhomerus*	LC	NT	/	237–1098	E, M, S	988	57.02%	23.77
Maritime striped squirrel *Tamiops maritimus*	LC	LC	/	310–1077	E, M, S	28	7.89%	0.67

**Table 2:** Estimated *β* (SE) coefficients for the optimal model for occupancy of mammalian species at the Jianfengling Branch of Hainan Tropical Rainforest National Park, China, from October 2020 to November 2021. *β* coefficients values that are significant at a level of 0.05 are in bold text.

Species	Covariates						
	Intercept	Elevation	NDVI	HFI	E	V	S
Common palm civet	−0.369 (0.203)	**0.518 (0.211)**	-	-	-	-	-
Hainan muntjac	−0.075 (0.218)	**1.298 (0.266)**	-	-	-	-	-
Mainland leopard cat	−0.546 (1.940)	**0.755 (2.720)**	-	-	-	-	-
Rhesus monkey	**−1.841 (0.597)**	**1.792 (0.680)**	0.294 (0.304)	-	**3.387 (1.198)**	17.896 (856.16)	**4.888 (2.035)**
Small-toothed ferret badger	0.056 (0.190)	0.359 (0.194)	-	-	-	-	-
Wild boar	**1.370 (0.237)**	−0.339 (0.239)	-	-	-	-	-
Northern treeshrew	**−1.290 (0.288)**	−0.300 (0.270)	-	-	-	-	-
Asiatic brush-tailed porcupine	0.200 (0.192)	0.279 (0.195)	−0.354 (0.205)	-	-	-	-
Malayan porcupine	**−3.120 (0.548)**	**-**	−0.680 (0.448)	-	-	-	-
Red-hipped squirrel	0.210 (0.195)	0.367 (0.203)	-	0.343 (0.210)	-	-	-

Note: NDVI: Normalized Difference Vegetation Index; HFI: human footprint index; E: tropical evergreen monsoon forest; S: tropical semi-deciduous monsoon forest; V: tropical valley rainforest.

## Data Availability

The data presented in this study are available upon request from the corresponding authors.

## Supplementary Materials

The following supporting information can be downloaded at: https://www.mdpi.com/article/10.3390/ani16050721/s1, Table S1: The occupancy models for each species in the Jianfengling Branch of Hainan Tropical Rainforest National Park, from October 2020 to November 2021; Table S2: Number of independent detections in the Jianfengling Branch of Hainan Tropical Rainforest National Park, China; Table S3: The detect and non-detect array data used in occupancy analysis.
