# Supplementary material for: Unveiling the Mammalian Diversity and Conservation Significance of Jianfengling Region: A Camera-Trapping Survey of Mammals in Hainan Tropical Rainforest National Park
Source: Animals (Basel). 2026 Feb 25;16(5):721. doi: 10.3390/ani16050721 (PMC12984735; doi:10.3390/ani16050721)
Supplement: Supplementary file 1 [file animals-16-00721-s001.zip › supplementary/Table S1 The occupancy models for each species in the Jianfengling Branch of Hainan Tropical Rainforest National Park, from October 2020 to November 2021..docx]

Table S1 The occupancy models for each species in the Jianfengling Branch of Hainan Tropical Rainforest National Park, from October 2020 to November 2021.

| Species | Covariates | ΔAIC | AICweight |
| --- | --- | --- | --- |
| Mainland leopard cat | elev | 0.00 | 0.33 |
|  | elev+veg | 1.25 | 0.18 |
|  | elev+NDVI | 1.57 | 0.15 |
|  | elev+Hii | 1.95 | 0.13 |
| Northern treeshrew | elev | 0.00 | 0.43 |
|  | elev+NDVI | 0.92 | 0.27 |
| Pallas's squirrel | elev | 0.81 | 0.16 |
|  | NDVI | 1.23 | 0.13 |
|  | Hii | 1.43 | 0.11 |
|  | elev+NDVI | 1.79 | 0.10 |
| Hainan muntjac | elev | 0.00 | 0.38 |
|  | elev+Hii | 1.28 | 0.20 |
|  | elev+NDVI | 1.96 | 0.14 |
| Red-hipped squirrel | elev+Hii | 0.00 | 0.24 |
|  | elev | 0.83 | 0.16 |
|  | Hii | 1.35 | 0.12 |
|  | elev+Hii+NDVI | 1.53 | 0.11 |
| Wild boar | elev | 0.00 | 0.22 |
|  | Hii | 1.968 | 0.081 |
|  | elev+NDVI | 1.970 | 0.081 |
|  | NDVI | 1.974 | 0.081 |
|  | elev+Hii | 2.00 | 0.080 |
| Swinhoe's striped squirrel | Hii | 0.56 | 0.21 |
|  | NDVI | 0.66 | 0.16 |
|  | elev | 01.31 | 0.15 |
|  | Hii+NDVI | 1.53 | 0.11 |
| Small-toothed ferret badger | elev | 0.00 | 0.24 |
|  | elev+veg | 1.77 | 0.10 |
|  | elev+NDVI | 1.84 | 0.095 |
|  | elev+Hii | 1.99 | 0.088 |
| Chinese pangolin | NDVI | 0.82 | 0.14 |
|  | Hii | 1.77 | 0.09 |
|  | elev+NDVI | 1.89 | 0.084 |
|  | elev | 1.90 | 0.084 |
| Malayan porcupine | NDVI | 0.00 | 0.13 |
|  | elev+NDVI | 0.35 | 0.11 |
|  | Hii+NDVI | 0.36 | 0.11 |
|  | veg+NDVI | 0.84 | 0.11 |
|  | Hii | 0.95 | 0.085 |
|  | elev | 1.12 | 0.081 |
|  | elev+Hii+NDVI | 1.45 | 0.074 |
|  | veg+Hii+NDVI | 1.49 | 0.063 |
| Asiatic brush-tailed porcupine | elev+NDVI | 0.00 | 0.21 |
|  | NDVI | 0.083 | 0.20 |
|  | elev | 1.184 | 0.11 |
|  | elev+Hii+NDVI | 1.891 | 0.081 |
| Rhesus monkey | elev+veg+NDVI | 0.00 | 0.95 |
| Common palm civet | elev | 0.00 | 0.57 |
|  | elev+Hii | 1.22 | 0.31 |
